# A robust genome assembly with transcriptomic data from the striped bark scorpion, *Centruroides vittatus*

**DOI:** 10.1093/g3journal/jkae120

**Published:** 2024-06-17

**Authors:** Tsunemi Yamashita, Douglas D Rhoads, Jeff Pummill

**Affiliations:** Department of Biological Sciences, Arkansas Tech University, Russellville, AR 72801, USA; Department of Biological Sciences, University of Arkansas-Fayetteville, Fayetteville, AR 72701, USA; High Performance Computing Center, University of Arkansas-Fayetteville, Fayetteville, AR 72701, USA

**Keywords:** Arachnid, scorpions, genome, scorpion toxin

## Abstract

Scorpions, a seemingly primitive, stinging arthropod taxa, are known to exhibit marked diversity in their venom components. These venoms are known for their human pathology, but they are also important as models for therapeutic and drug development applications. In this study, we report a high-quality genome assembly and annotation of the striped bark scorpion, *Centruroides vittatus*, created with several shotgun libraries. The final assembly is 760 Mb in size, with a BUSCO score of 97.8%, a 30.85% GC, and an N50 of 2.35 Mb. We estimated 36,189 proteins with 37.32% assigned to Gene Ontology (GO) terms in our GO annotation analysis. We mapped venom toxin genes to 18 contigs and 2 scaffolds. We were also able to identify expression differences between venom gland (telson) and body tissue (carapace) with 19 sodium toxin and 14 potassium toxin genes to 18 contigs and 2 scaffolds. This assembly, along with our transcriptomic data, provides further data to investigate scorpion venom genomics.

## Introduction

Scorpions are an ancient and diverse arthropod taxa primarily known for their medical importance and seemingly little morphological change over millions of years (Sharma *et al.* 2015; Lourenço 2018). Although all scorpions have a similar bauplan, they show immense variation in their venom components (Sunagar *et al.* 2013; Sharma *et al.* 2015; Housley *et al.* 2017; Lourenço 2018). The scorpion genus *Centruroides* constitutes the most medically important and one of the most diverse and wide-ranging scorpion taxa in North America (Gantenbein *et al.* 2001; Santibáñez-López *et al.* 2016; Esposito and Prendini 2019). The complex evolutionary history and geographic variability of this genus have generated controversy and taxonomic confusion (Sissom 1990; Borges *et al.* 2012; Santibáñez-López *et al.* 2016). The diversity of the venom also implies remarkable evolutionary adaptations with new and varied constituents discovered annually (Santibáñez-López *et al.* 2016; Housley *et al.* 2017). In spite of their medical importance, scorpion genomics has lagged behind venom transcriptomics and proteomics, with only 3 of the estimated 2,500 worldwide scorpion species having genome assembly entries in the NCBI database (Santibáñez-López *et al.* 2022).

**Fig. 1.**
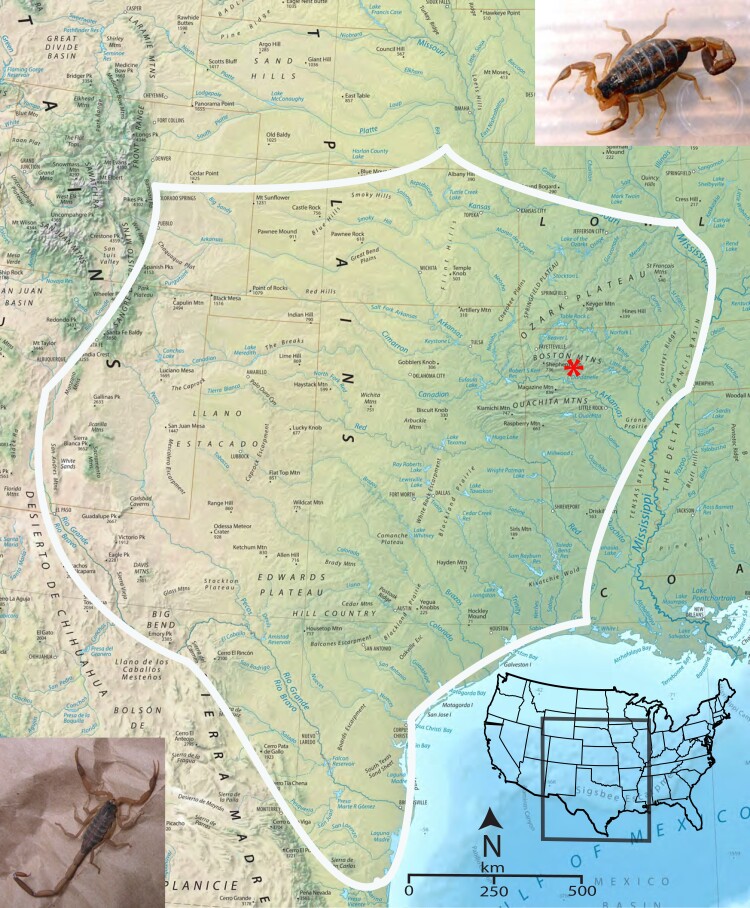
An approximate geographic range of *C. vittatus*, with a red asterisk identifying the location for the individuals collected for the genomic analysis [modified from Yamashita and Rhoads (2013)]. The photograph in the lower left inset shows a male scorpion and the upper right inset shows a female.

The scorpion *Centruroides vittatus* encompasses a large geographic range across the Western USA and Northern United Mexican States (Fig. 1). Although a member of the toxic *Centruroides* genus, this species is not known as medically important (Kang and Brooks 2017). However, due to a coevolution with mammalian predators, evidence suggests that western *C. vittatus* populations may possess a more medically significant venom than eastern populations (Rowe and Rowe 2008; Bowman *et al.* 2021). Throughout its geographic range, *C. vittatus* is commonly found in diverse ecological habitats, but populations across the northern and eastern geographic distributions appear to prefer dry, rocky south-facing slopes or glade areas. The human introduction of this scorpion appears to also have created additional populations outside its known geographic range (Shelley and Sissom 1995). In this study, we present the assembled, annotated genome of *C. vittatus*. The integration of genome and transcriptome data shows novel splicing and transcriptional activity around venom gene regions. Furthermore, this genome will complement the deposited genome of the more noxious western *Centruroides sculpturatus* and expand on the analysis of this ancient taxon.

## Materials and methods

### Genome sequencing and assembly

Total genomic DNA was extracted from 4 scorpions collected in Pope County, AR, with the Qiagen genomic tip and genomic DNA buffer set (Qiagen, Inc.). The genomic DNA quality and quantity were analyzed through 0.9% agarose gel electrophoresis, Qubit, and UV spectroscopy. One female genomic DNA sample was sent to the University of Arkansas for Medical Sciences DNA Sequencing Core Facility for 300 base paired–end sequencing on an Illumina MiSeq. Two genomic DNA samples (1 male and 1 female) were sent to the National Center for Genome Resources (NCGR, NM) for PacBio 20K library generation and sequencing on 10 SMRT cell for each individual genome (Supplementary Tables 1 and 2). A final female sample was sent to the CTPR Genomics Lab at Arkansas Children's Hospital Research Institute for a 300-cycle mid-output Illumina NextSeq genome sequencing.

The de novo assembly was conducted at the Arkansas High Performance Computing Center at the University of Arkansas. Sequence read data quality control check for Illumina short reads was conducted with FastQC (0.11.5; Andrews 2010) and trimmed with default settings in Trimmomatic (0.39; Bolger *et al*. 2014). For PacBio CLR long reads, NanoPlot (1.0.0; De Coster *et al*. 2018) and FiltLong (0.2.0; Wick 2018) were utilized. The MiSeq reads were incorporated into the Flye assembly to correct the long PacBio reads. The 2 PacBio CLR long reads and the Illumina NextSeq quality trimmed reads were assembled with several software tools: MaSuRCA (V3.4.0; Zimin *et al.* 2013), Flye (V2.8.1; Kolmogorov *et al.* 2019), and also a version that was error-corrected using Ratatosk (V0.1; Holley *et al.* 2021) and the Illumina data before assembly, with Flye also utilizing consensus polishing via the tool incorporated with Flye. Draft assemblies were evaluated by following 2 criteria: (1) the N50 statistic from contigs' size, using QUAST v.5.0.2 (Gurevich *et al.* 2013), and (2) the completeness score based on the presence of universal single-copy ortholog genes, using BUSCO v.4.1.0 (Manni *et al.* 2021) against Arachnida ortholog dataset 10 (arachnida_odb10). Lastly, we identified and removed a unique *Mycoplasma* genome from our reads (Yamashita *et al.* 2019). The *Mycoplasma* genome was identified from the PacBio genomic sequence assembly as a unique 683,827 bp contig with a distinct GC content (43.7%) compared with the 30.85% GC content calculated for the scorpion contigs.

### Transcriptome assembly and annotation

Two male scorpions and 1 female scorpion were collected in northwest Arkansas, fed crickets with a visual conformation of prey envenomation, then after 3 days, harvested for telson (venom gland) and carapace (body tissue) transcriptome analysis. The scorpions were flash-frozen at −80°C and total RNA extracted with a Trizol preparation (Sigma-Aldrich, St Louis, MO, USA). RNA sample qc was analyzed through electrophoresis with an Aligent TapeStation system. RNA-seq with 50 bp reads was conducted at the University of Delaware on an Illumina genome sequencer (Illumina, Inc., San Diego, CA, USA) as described in Van Every and Schmidt (2021). The data were viewed for initial quality through FastQC (v0.11.7), trimmed with Trimmomatic (v0.36; Bolger *et al.* 2014), and normalization of the data was performed using Trinity (v2.5.1; Haas *et al.* 2013). Assembly of the normalized reads was then performed with the following de novo assembly programs: Trinity (v2.5.1), SOAPdenovo2 (v2.4.1; Li *et al.* 2009), Velvet (v1.2.10; Zerbino and Birney 2008), and TransAbyss (v1.5.4; Robertson *et al.* 2010), resulting in 4 individual assemblies. The transcriptome assemblies were then aggregated together using EviGene (Gilbert 2019), to remove redundancies, pick the best representatives, and filter out misassemblies. The trimmed reads were mapped to the genome assembly using NGen and quantified as RPKM using ArrayStar. RPKMs for contigs identified by BLASTn of the assembly for key genes were extracted for each sample over all contigs matching that BLAST query. In addition, the transcriptome assembly was blasted with a query scorpion toxin database created from the NCBI scorpion toxin and our current sodium toxin databases (2,133 total toxin sequences). From these BLAST searches, RPKM values for the 2 males and one female were summarized for sodium toxin RNAs with additional searches for additional scorpion toxin RNAs. In a previous study, the transcriptome data were included with RT-qPCR and venom protein mass spectrophotometry data to quantify sodium β-toxin gene expression and toxin protein variation in this scorpion species (Bowman *et al.* 2021).

### Genome annotation

Repetitive elements were catalogued using RepeatModeler (V2.0.2a; Flynn *et al.* 2020) and repetitive elements masked with RepeatMasker (V4.1.2, Smit *et al*. 2013). The repeat masked genome was indexed, and RNA-seq reads from the carapace (body tissue) and the telson (venom gland) were aligned with STAR (V2.7.9a; Dobin *et al.* 2013) to create BAM files. Additionally, the RNA-seq data were utilized to annotate the repeat masked scorpion genome with BRAKER (V2.1.6; Hoff *et al.* 2019). The predicted proteins from BRAKER were then implemented in a BLAST analysis of the TrEMBL database. Lastly, the polished *C. vittatus* genome with carapace and telson RNA-seq BAM files, the predicted, annotated polypeptides from BRAKER, and the TrEMBL BLAST were loaded into Integrative Genomic Viewer (IGV; V2.13.2a; Robinson *et al.* 2011) to view RNA-seq Sashimi plots of the expression data relative to annotated exons. We also built a toxin BLASTn database with the polished *C. vittatus* genome against a scorpion toxin query file housing 2,133 sequences to further map toxin genes. The IGV visualizations to examine differential expression between carapace and telson were focused on contigs and scaffolds containing putative toxin genes based on the BLASTn toxin queries.

A functional annotation analysis used the Cyverse pipeline developed for arthropods (Saha *et al*. 2021). This pipeline combines outputs from Gene Ontology (GO) annotations (GOanna) and InterProScan (functional motifs) as well as mapping proteins to pathways via KOBAS.

## Results and discussion

### Genome assembly and annotation

**Fig. 2.**
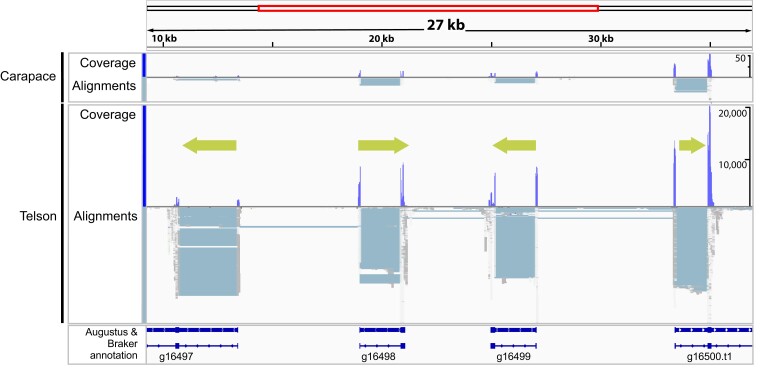
Integrative genomic viewer visualization of contig 1,491 (49 kb) showing putative sodium toxin gene expression mapped to show coverage and read alignments with respect to carapace (body tissue) and telson (venom gland). Our scorpion toxin BLAST results identified this sodium toxin as the alpha toxin Cn12 like (XP_023242001.1) identified in the *C. sculpturatus* genome and suggests a clustering of sodium toxin genes in this contig. The arrows correspond to gene orientation. The section of the contig that shows the 4 putative sodium toxin gene regions spans 27 kb with a intervening genome sequence of 3–8 kb. Predicted proteins from the Augustus and BRAKER results are mapped in the bottom panel.

**Fig. 3.**
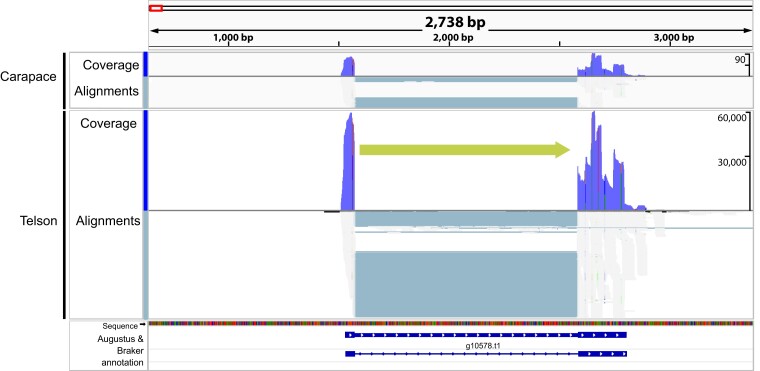
Integrative genomic viewer visualization of contig 2703 (157 kb) showing putative sodium toxin gene expression mapped to show coverage and read alignments with respect to carapace and telson. Our BLAST results identified this sodium toxin as the sodium channel blocking toxin from *C. noxius*. The green arrow corresponds to gene orientation. The section of the contig that shows the putative sodium toxin gene region spans 2,150 bp. Predicted proteins from the Augustus and BRAKER results are mapped in the bottom panel.

**Fig. 4.**
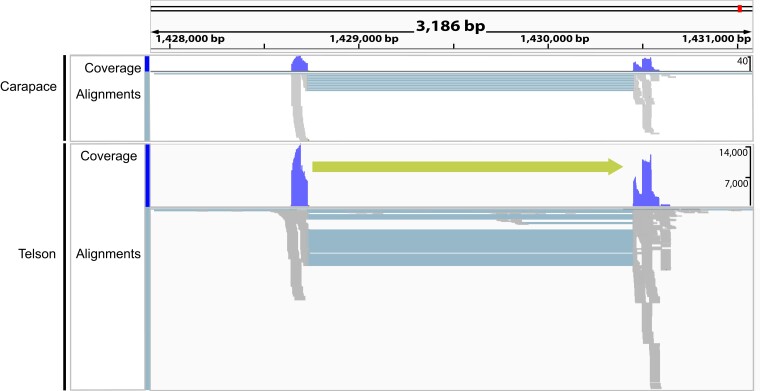
Integrative Genomic Viewer visualization of contig 82 (1,461 kb) showing putative potassium toxin (ERG1) gene expression mapped to show coverage and read alignments with respect to carapace and telson. The green arrow corresponds to gene orientation. The predicted proteins are not shown, as none were mapped to this region.

The 4 genome sequencing outputs resulted in 3 final de novo *C. vittatus* genome assemblies (Table 1). Compared with the 2 other scorpion genomes available, *C. vittatus* exhibited a smaller genome size (760 Mb) to the *C. sculpturatus* (926.4 Mb) and *Mesobuthus martensii* (925.5 Mb) genomes (Garb *et al.* 2018). Of the 3 final genome assemblies, the Ratatosk–Flye assembly was judged as the most complete as it exhibited the largest reduction in contig number, with the largest contig size and N50 (Table 1, Supplementary Table 1). This assembly also showed the best BUSCO statistics with 97.8% complete (92.6% unique, 5.2% duplicates; Table 2). The transcriptome basic statistics for the venom gland are presented in Table 3 with the repetitive element data in Supplementary Table 2. The transcriptomic data and the annotated polypeptide data were incorporated into IGV to visualize gene expression variation between the venom gland and body tissue (Figs. 2–4). The functional annotation workflow predicted 36,189 proteins, which was higher than the 17,364 proteins predicted in *Latrodectus hesperus* (Western Black Widow Spider; Saha *et al.* 2021) but comparable to the *C. sculpturatus* protein number of 35,529. The GOanna and InterProScan results between *C. vittatus* and *L. hesperus* were comparable (Table 4). The KOBAS output showed a marked difference, with *C. vittatus* exhibiting higher numbers of proteins assigned to pathways and percent assigned to KEGG pathways when compared with *L. hesperus* (Table 5).

**Table 1.** Assembly statistics from 3 assembled genomes of *C. vittatus* and *C. sculpturatus*.

Quast assembly statistics	MaSuRCA	Flye	Ratatosk–Flye	*C. sculpturatus*
Number of contigs (≥0 bp)	6,383	2,842	2,071	
Number of contigs (≥50,000 bp)	5,787	2,177	1,694	
Total length (≥0 bp)	791,048,345	769,382,264	760,848,696	925,474,958
Number of contigs	6,135	2,440	1,837	35,614
Largest contig	2,437,683	10,350,238	11,744,082	
Total length	790,486,062	768,752,645	760,461,457	863,133,193
GC (%)	30.89	30.85	30.85	31.4
N50	336,247	1,876,222	2,358,559	

The MaSuRCA assembly incorporates the aggregated PacBio CLR long reads and the Illumina NextSeq reads. The Flye assembly only incorporates the aggregated PacBio CLR long reads, and the Ratatosk–Flye assembly incorporates the aggregated PacBio CLR long reads with Ratatosk correction with MiSeq short reads.

**Table 2.** BUSCO statistics of the 3 *C. vittatus*–assembled genomes.

BUSCO statistics	MaSuRCA	%	Flye	%	Ratatosk–Flye	%
Complete BUSCOs (C)	2,794	95.2	2,842	96.8	2,870	97.8
Complete and single-copy BUSCOs (S)	2,615	89.1	2,686	91.5	2,716	92.6
Complete and duplicated BUSCOs (D)	179	6.1	156	5.3	154	5.2
Fragmented BUSCOs (F)	23	0.8	26	0.9	14	0.5
Missing BUSCOs (M)	117	4.0	66	2.3	50	1.7
Total BUSCO groups searched	2,934		2,934		2,934	

BUSCO Lineage data set for BUSCO statistics: arachnida_odb10.2019-11-20 lineage markers.

**Table 3.** Transcriptome statistics from a combined *C. vittatus* telson (toxin gland) of 3 scorpions.

Transcript number	226,162
Transcripts >500 bp	48,463
Transcripts >1,000 bp	26,909
Average length of assembled transcripts (bp)	463,164
Longest transcript (bp)	9,430
Total length (bp)	104,750,193
Transcript N50	1,149

From Bowman *et al*. (2021).

**Table 4.** Functional annotation analyses with Goanna and InterProScan results.

			GOanna (BLAST)		InterProScan (motif analysis)	
Species	Proteins	Proteins assigned GO terms	Proteins assigned GO	Average GAQ	Proteins assigned GO	Average GAQ
*Centruroides vittatus*	36,189	37.32%	7.37%	—	30.45%	—
*Latrodectus hesperus*	17,364	31.17%	2.02%	197.44	30.44%	28.896

The *C. sculpturatus* protein number is estimated as 35,529.

**Table 5.** Functional annotation analyses with KOBAS results with program parameters noted in the footnotes.

		All pathways		KEGG pathways	
Species	Proteins	Proteins assigned to pathways	Average number of proteins in pathways	% Assigned to pathways	Average number of proteins in pathways
***Centruroides vittatus***	36,189	63.99%	8.00	81.29%	35.92
***Latrodectus hesperus***	17,364	30.06%	4.06	16.97%	22.66

Databases: PANTHER, Gene Ontology Slim, KEGG PATHWAY, Gene Ontology. Statistical test method: hypergeometric test/Fisher's exact test. FDR correction method: Benjamini and Hochberg.

### Toxin gene specifics

The BRAKER annotation of the Ratatosk–Flye assembly initially mapped putative toxin genes to 2,011 contigs and 60 scaffolds (Table 6, Supplementary Table 3). The mapping of toxin genes to the assembly with the scorpion toxin BLASTn file refined the genes to a subset of 848 contigs and 57 scaffolds. A further analysis and refinement of the BLASTn output identified putative toxin genes in 18 contigs and 2 scaffolds in which the toxin genes showed a much higher expression in the telson (venom gland) vs the carapace, including 19 putative sodium and 14 putative potassium toxin genes.

**Table 6.** Scorpion toxin gene expression mapped to the *C. vittatus* genomic assembly.

	Toxin genes and percentages mapped to contigs	Toxin genes and percentages mapped to scaffolds
**Ratatosk–Flye** a**ssembly** t**otals**	2011	60
**Scorpion** t**oxin BLASTn file**	846 (42%)	57 (95%)
**Filtered** s**corpion BLASTn Na/K results with a higher telson expression than carapace**	17 Na toxins and 14 K toxins to 18 contigs	2 Na toxins to 2 scaffolds

The Ratatosk–Flye assembly totals represent the subset of contigs and scaffolds with identified putative toxin genes from BRAKER. The scorpion toxin BLASTn file represents contigs and scaffolds with toxin gene hits from a query file of 2,133 scorpion toxin genes. The total sodium and potassium toxin genes with a higher telson expression mapped to the 18 contigs and 2 scaffolds from the filtered BLASTn data are 19 and 14 genes, respectively.

The toxin gene mapping suggests that many toxin genes are only differentially expressed in body tissue vs venom glands, rather than uniquely expressed in venom glands. One contig that spans 35 kb (contig 1491) shows 5 paralogs of a putative sodium toxin gene in tandem, separated by intervening genomic sequences of 3–8 kb, suggesting ancestral gene duplication in this region (Fig. 2). The IGV view of contig 1491 also suggests that the sodium toxin genes in this region are arranged on both + and − DNA strands, which may indicate gene inversions.

Other contigs showed a pattern of larger genomic regions with mapped sodium toxin genes. For example, contig 2703 shows a 1,900 bp region with putative sodium toxin genes mapped, suggesting multiple sodium toxin genes in this region (Fig. 3). Putative potassium toxin genes were located on other contigs and scaffolds, 6 vs 15 for putative sodium toxin genes, with no evidence of duplicated regions (e.g. Fig. 4). These genes also appear to exhibit differential expression in the telson vs the carapace rather than unique expression in the telson. These initial findings support a model of recent toxin gene duplication events that may underline the incredible sodium toxin diversity in the new world *Centruroides* species (Rendón-Anaya *et al.* 2012; Drukewitz and von Reumont 2019).

## Conclusion

We describe a genomic assembly and annotation for the scorpion *C. vittatus* coupled with transcriptomic data mapped to contigs and scaffolds. Our assembly shows a genome of 760 Mb in length, with 98% of sequences mapped to 2,071 contigs. Our results also highlight the substantial toxin gene diversity in this scorpion and show toxin gene expression patterns between body tissue and the venom gland. This genome will complement the growing number of venomous species with genomes in published databases.

## Supplementary Material

jkae120_Supplementary_Data

## Data Availability

The genome assembly was deposited at the NCBI under accession number JASCZU000000000; BioProject PRJNA937744; BioSample SAMN33417986. Transcriptome datasets were deposited in the NCBI with the following IDs: TSA: GIPT01000000, SRA: SRR11917465, BioProject: PRJNA636371, BioSample: SAMN15075759. The datasets entered into IGV (carapace and telson bam files with the scorpion BLASTp file) and the parameters for the genome assemblies were deposited into the following github site: https://github.com/TsuYamashita/C_vittatus-RNAseq-Data.git. Supplemental material available at G3 online.
