# Supplementary material for: A robust genome assembly with transcriptomic data from the striped bark scorpion, Centruroides vittatus
Source: G3 (Bethesda). 2024 Jun 17;14(8):jkae120. doi: 10.1093/g3journal/jkae120 (PMC11304958; doi:10.1093/g3journal/jkae120)
Supplement: jkae120_Supplementary_Data [file jkae120_supplementary_data.pdf]

Supplementary Information

Table S1. Initial genome sequencing results from PacBio genome sequencing.

Job Metric	Q1133	Q1171
Number of Bases	6,812,411,387	9,669,253,726
Number of Reads	555,183	642,400
N50 Read Length	18,378	21,548
Mean Read Length	12,270	15,051
Read Quality Score	0.85	0.85

Table S2. Repetitive elements in the *C. vittatus* genome identified from a RepeatMasker output.

Identity	Number of elements	Length occupied (bp)	Percentage of sequence (%)
Retro elements	68,527	46,749,745	6.14
Penelope	28,475	12,237,284	1.61
LINEs	59,660	37,732,270	4.96
R1/LOA/Jockey	10869	10,675,340	1.4
LTR elements	8,867	9,017,475	1.19
DNA transposons	131,005	52,169,258	6.86
Tc1-IS630-Pogo	69,372	25,562,168	3.36
Unclassified	931,189	214,632,422	28.21
Total Interspersed repeats		313,551,425	41.21
Simple repeats	268,925	11,706,345	1.54

***Only elements above 1% are reported.

Table S3. Putative *C. vittatus* Sodium Beta Toxin polypeptide sequence diversity from a survey of 10 population groups identified in a *C. vittatus* phylogeographic analysis. Also included are CsEv3b and CvIV4 toxin sequences and additional *C. sculpturatus* Beta toxin sequences obtained through a Cloning experiment. Cysteine residues are highlighted in green. The sodium toxin gene sequences for these polypeptides were included in the toxin BLASTn database. For additional information, see Bowman et al. (2021).

```
                    1        10        20        30        40        50        60        70        80        90        100
                    |         |         |         |         |         |         |         |         |         |         |
          CsEv3b    KEGYLVNKSTGCKYGCLKLGENEGCDKECKAKNQGGSYG-YCY-AFACWCEGLPESTPTYPLPNKSCGKK---------------------------------------
           CvIV4    KKDGYPVEHS-GCKYTCWK---NEYCDKV-CK--DLKGEGG-YCYINLTCWCTGLPDNVPLKT--NQRCNGKRK-----------------------------------
     CsCNaTBet01    REGYLVRKSDNCKHGCIPGIDEDYCGDICKKRNRGGKKGWCQKYGCWCTGMPESTQTYPIPGKPCSD------------------------------------------
     CsCNaTBet02    KEGYLAKLLTRQRRHSFEDRELPLFRDSENAGQLFNSDGEQDVGNPLLASWGKLGRDYIYLLSDLESSQELDAFVDVTPDNPPSLSCRH--------------------
     CsCNaTBet03    KEGYLAKLLTRQRRHSFEDRELPLFRDSENAGQLFNSDGEQDVGNPLLASWGKLGRDYIYLLSDLESSQELDAFVDVTPDNPPSLSCRR--------------------
     CsCNaTBet05    REGYLVRKSDNCKHGCIPGIDEDYCGDICKKRNRGGKKGWCQKYGCWCTGMPESTQTYPIPGKPCSD------------------------------------------
     CsCNaTBet06    KEGYLAIRGCIYSCVTDRECETECKKYGGKIGFCRFLCYCEGLPKSVPTLPKPGKKCAIPGFSSITSEFAAACRSTIWESSQRVGCIA---------------------
 CviNaTBet_BCR_01   KEGFLALRGCYAVV*RIGSAKQNARSTEEKLDFADFLRAIAKVCPKVYRLCPNLVENAPYRASP---------------------------------------------
 CviNaTBet_BCR_02   KEGFLAMRGCYTVA*RIGSAKQNARSTEEKLDFADFLRAIAKVCPKVYRLCPNLVENAPYRASP---------------------------------------------
 CviNaTBet_BCR_03   PEGWDAYLECSIVPPK*RIGSAKQNARSTEEKLDFADFLRAIAKVCPKVYRLCPNLVENAPYRASP-------------------------------------------
 CviNaTBet_BCR_04   KEGYLAIRGCIYSCVTDRECETECKKYGGKIGFCRFLTCYCEGLPKSVPTVPKPGRKCAIPGFS---------------------------------------------
 CviNaTBet_BCR_05   KEGYLAIRGCIYSCVTDRECETECKKYGGKIGFCRFLTCYCEGPPKSVPTVPKPGRKCAIPGFSSITSEFAAACRSTIWESSQRVGCIA--------------------
 CviNaTBet_BCR_06   KDGYLALRGCNAVA*RIGSAEQNARSTEEKLDFADFLRAIAKVCPKVYRLCPNLVENAPYRASP---------------------------------------------
 CviNaTBet_BCR_07   KDGILP*GFYNAAA*RIGSAKQNARSTEEKLDFADFLLAIAKVCPKVYRLCPNLVENAPYRASP---------------------------------------------
 CviNaTBet_BCR_08   KEGYLVNKKTGCKYNCMLKIGDSHCDKECKAPNQGGSYGYCFKLGCWCEGLPESTPTYPLPDKSCSG------------------------------------------
 CviNaTBet_BCR_09   REGYLVNKKT---------------------------------------------------------------------------------------------------
 CviNaTBet_BCR_10   KEGYLVKKSDGCKYGCVMLFGDSNCDMECKAPNQGGQKGWCYAFACWCTGMPESAQVYPSDKSCGKK------------------------------------------
 CviNaTBet_BCR_11   KEGYLVKKSDNCKHGCIPGFDEDYCDDICKTRNRGGKKGWCQKYGCWCTGMPESTQTYPIPGKPCSS------------------------------------------
 CviNaTBet_BCR_12   REGYQISGSDLAPNPVTQQLMNLGATIYFNHRPENVRDASVVVVSSAISADNPPL------------------------------------------------------
 CviNaTBet_BCR_13   KEGYLVNKKT---------------------------------------------------------------------------------------------------
 CviNaTBet_BCR_14   -------------------------KEGYLAKNQGGEKG-WCK-NFGCWCTGMPESTPTWPI-GKPCSGK---------------------------------------
 CviNaTBet_BCR_15   KEGYLAKKGDGCKYGCTPYFGDEGCDKECKAKNQGGEKG-WCK-NFGCWCTGMPESTPTWPI-GKPCSGK---------------------------------------
  CviNaTBet_HT_01   KEGYLVNKSDGCKYGCVMLIGDEGCNKECKAPNQGGTKGWCYAFGCWCTGMPESTQVYPLPNKSCGKK-----------------------------------------
  CviNaTBet_HT_02   REGYLAIRGCIYSCVTDRECETECKKYGGKIGFCRFLTCYCEGLPKSVPTVPKPGRKCA--------------------------------------------------
  CviNaTBet_HT_03   REGYLVNKSDGCKYGCVMLIGDEGCNKECKAPNQGGTKGWCYAFGCWCTGMPESTQVYPLPNKSCGKK-----------------------------------------
  CviNaTBet_HT_04   REGYLVRKSSNCKDGCIPGIDEDYCDDRCKARNQGGKKGWCQKYGCWCTGMPESTQTYPIPGKPCSD------------------------------------------
  CviNaTBet_HT_05   REGYLAIRGCIYSCVTDRECETECKKYGGKIGFCRFLTCYCEGLPKSVPTLPKPGRKCA--------------------------------------------------
  CviNaTBet_HT_06   KEGYLAIRGCIYSCVTDRECETECKKYGGKIGFCRFLTCYCEGLPKSVPTLPKPGRKCA--------------------------------------------------
  CviNaTBet_HT_07   REGYLYAVGGNDGSSSLNTVERYNPRHNKWMLVTSMTLRRSSVGVAVVDSPCMELILTRKSGQ----------------------------------------------
  CviNaTBet_HT_08   REGYLVNKKTGCKYGCTPKLGDDHCDKECKAPNQGGKKGWCKNFGCWCTGMPESTQTWPIPGKSCSS------------------------------------------
  CviNaTBet_HT_09   REGYLVKKSDGCKYGCTPKLGDDHCDKECKAPNQGGKKGWCKNFGCWCTGMPESTQTWPIPGKSCSR------------------------------------------
CviNaTBet_Chin_01   KEGYLVKKSDGCKYGCVMLIGDSNCDMECKAPNQGGQKGWCYAFGCWCTGMPESTQVYPLPGKSCGKK-----------------------------------------
CviNaTBet_Chin_02   KEGYLVKKSDGCKYGCVMLIGDSNCDMECKAPNQGGQKGWCYAFACWCTGMPESAQVYPS-DKSCGKK-----------------------------------------
CviNaTBet_Chin_03   REGYLA--IRGCIYSCV---TDRECETECK--KYGGKIGFCRFLTCYCEGLPKSVPTLPKPGRKCA-------------------------------------------
CviNaTBet_Chin_04   REGYLVKKSDGCKYGCVMLIGDSNCDMECKA------------------------------------------------------------------------------
CviNaTBet_Chin_05   KEGYLVRKSDNCKHGCIPGIDEDFCDDICKARNRGGKKGWCQKYGCWCTGMPESTQTYPIPGKPCSS------------------------------------------
CviNaTBet_Chin654   KEGYLAIKTTCCKYTRMT---DRVCIAECK--KNGGKTG-FCQ-FLGCYCLDLPESAPTWPIHGKPCSGK---------------------------------------
CviNaTBet_AgSp_01   REGYLAIRGCIYSCVADRECETECKKYGGKTGFCRFLTCYCEGLPKSVPTLPKPGRKCA--------------------------------------------------
CviNaTBet_AgSp_02   KEGYLAIRGCIYSCVTDRECETECKKYGGKIGFCRFLTCYCEGLPKSVPTVPKPGRKCA--------------------------------------------------
CviNaTBet_AgSp_04   KEGYLVKKSDNCKHGCIPGFDEDYCDDICKTRNRGGKKGWCQKYGCWCTGMPESTQTYPIPGKPCSS------------------------------------------
CviNaTBet_AgSp_05   REGYLVNKSDGCKYGCVMLIGDEGCNKECKAPNQGGTKGWCYAFGCWCTGMPESTQVYPLPNKSCGKK-----------------------------------------
CviNaTBet_AgSp667   KEGYLAIR--GCIYSCVT---DRECETECK--KYGGKIG-FCR-FLTCYCEGLPKSVPTVPKPGRKCA-----------------------------------------
CviNaTBet_AgSp668   KEGYLVKKSDGCKYGCTPYFGDEGCDKECKAKNQGGEKG-WCK-NFGCWCTGMPESTPTWPI-DKTCSKK---------------------------------------
CviNaTBet_AgSp_06   REGYLVKKSDNCKHGCIPGFDEDYCD-DICKTRNRGGKKG-WCQ-KYGCWCTGMPESTQTYPIPGKPCSS
CviNaTBet_AgSp_07   REGYLAIR--GCIYSCVA---DRECETECK--KYGGKTG-FCR-FLTCYCEGLPKSVPTLPKPGRKCA
CviNaTBet_AgSp_08   KEGYLVKKSDNCKHGCIPGFDEDYCD-DICKTRNRGGKKG-WCQ-KYGCWCTGMPESTQTYPIPGKPCSS---------------------------------------
CviNaTBet_AgSp_09   REGYLVNKSDGCKYGCVMLIGDEGCNKECKAPNQGGTKG-WCY-AFGCWCTGMPESTQVYPLPNKSCGKK---------------------------------------
CviNaTBet_AgSp_10   KEGFWRSGAVIQLRDG*GVLNRLQEVRRKNWILPIFTCYC*GLPKSVPTVPKPGRKCAIPGFSSITIEFAAACMSTLWESSQRVGCIA
CviNaTBet_AgSp_11   KEGYLAMRGCIYSCVTDRECETECKKYGGKIGFCRFLTCYCEGLPKSVPTVPKPGRKCAIPGFSSITSEFAAACRSTIWESSQRVGCIA
 CviNaTBet_LBR_01   REGYLVKKSDGCKYGCVMLIGDSNCDMECKAPSQGGQKGWCYAFGCWCTGMPEGTQVYPLPGKSCGKK----------------------------------------
 CviNaTBet_LBR_02   REGYLVKKSDGCKYGCVMLIGDSHCDTECKAPNQGGKKGWCYALGCWCTGMPESTQVYPLPDKSCGKK----------------------------------------
 CviNaTBet_LBR_03   REGYLVNKSDGRKYGCVMLIGDEGCNKECKAPNQGGTKGWCYAFGCWCTGMPESTQVYPLPNKSCGKK----------------------------------------
 CviNaTBet_LBR_04   KEGYLVRKSDNCKDGCIPGIDEDYCDDRCKARNQGGKKGWCQKYGCWCTGMPESTQTYPIPGKPCSS-----------------------------------------
 CviNaTBet_LPb_01   KEGYQVNKSDGCKYGCVMLIGDEGCNKECKAPNQGGTKGWCYAFGCWCTGMPESTQVYPLPNKSCGKK----------------------------------------
 CviNaTBet_LPb_02   KEGYLAIRGCIYSCLTDRECETECKKYGGKIGFCRFLTCDCEGLPKSVPTVPKPGRKCA-------------------------------------------------
CviNaTBet_LPb3066   REGYLVKKSDGCKYGCVMLIGDSHCDTECKAPNQGGKKGWCYALGCWCTGMPESTQVYPLPDKSCGKK----------------------------------------
 CviNaTBet_LPb_03   KEGYLVNKKTGCKYNCMLKIGDSHCDKECKAPNQGGSYGYCFKLGCWCEGLPESTPTYPLPDKSCSG-----------------------------------------
 CviNaTBet_LPb_04   REGYLVNKKTGCKYNCMLKIGDSHCDKECKAPNQGGSYGYCFKLGCWCEGLPESTPTYPLPDKSCSG-----------------------------------------
 CviNaTBet_BMe_01   KEGYLALR--GCIYSCVT---DREYEAECK--KIGGKIGIWC--FFTSSCEGMSKGGQPVPQLGR-------------------------------------------
CviNaTBet_PaDu_01   KEGCLALRGLYTVG*RIGSAEQNARVRIKNWILPISYLLLLGSAQNSPTLPKLGRKCAIPGFSSITIEFAATCRPPLWESSQRVGCIPCLFYSV**R-----------
CviNaTBet_PaDu_01   KEGYLAIRGCIYSCVTDRERETECKKYGGKIGFCRFLTCYCEGLPKSVPTLPKPGRKCAIPGFSSITSEFAAACRSTIWESSQRVGCIA-------------------
CviNaTBet_PaDu_02   KEGFLAMRGCSTVA*RIGSAKQNARSTEEKLDFADFLRAIAKVCPKVYRLCPNLVENAPYRASPQSLVNSRPPAGRPYGRAPNALDA---------------------
CviNaTBet_PaDu_03   KEGYLVNKSDGCKYGCVMLIGDEGCNKECKAPNQGGTKGWCYAFGCWCTGMPESTQVYPLPNKSCGKK----------------------------------------
CviNaTBet_PaDu_04   KEGYLAIRGCIYSCVTDRECETECKKYGGKIGFCRFLTCYCEGLPKSVPTVPKPGGKCA-------------------------------------------------
CviNaTBet_PaDu_05   KEGYLVNKSNGCKYGCTPKLGDDHCDKECKARNQGGKKGWCKNFGC--------------------------------------------------------------
```

```
CviNaTBet_Kisa_01   KEGYLVNKSDGXIRLRDVDWRRGLQ-----------------------------------------------------------------------------------
CviNaTBet_Kisa_02   KEGYLVRKSDNCKHGCIPGIDEDFCDDICKARNRGGKKGWCQKYGCWCTGMPESTQTYPIPGKSCSS-----------------------------------------
CviNaTBet_Kisa_03   KEGYLVKKSDGCKYGCTPYFGDEGCDKECKAKNQGGEKGWCKNFGCWCTGMPESTPTWPI-DKTCSKK----------------------------------------
CviNaTBet_Kisa_04   REGYLVKKSDGCKYGCVMLIGDSNCDMECKAPSQGGQKGWCYAFGCWCTGMPEGTQVYPLPGKSCGKK----------------------------------------
CviNaTBet_Kisa_05   KEGYAIRGCIYSCVTDRECETECKKYGGKIGFCRFLTCYCEGLPKSVPTVPKPGRKCA--------------------------------------------------
  CviNaTBet_KS_01   KEGFWRSGAVIQLRDG*GVLNRLQEVRRKNWILPIFTCYC*GLPKSVPTVPKPGRKCAIPGFSSITIEFAAACMSTLWESSQRVGCIA*VFYSVT*IA----------
  CviNaTBet_KS_02   KEGYLAMRGCIYSCVTDRECETECKKYGGKIGFCRFLTCYCEGLPKSVPTVPKPGRKCAIPGFSSITSEFAAACRSTIWESSQRVGCIA-------------------
  CviNaTBet_KS_03   KEGYLAIRGCIYSCVTDRECETECKKYGGKIGFCRFLTCYCEGLPKSVPTVPKPGRKCAIPGFSSITSEFAAACRSTIWESSQRVGCIA-------------------
  CviNaTBet_KS_04   KEGYLAIRGCIYSCVTDRECETECKKYGGKIGFCRFLTCYCEGLLKSVPTVPKPGRNAPYRASPQSLVNSRPPAGRPYGRAPNALDA---------------------
  CviNaTBet_KS_05   REGYLVNKKTGCKYNCMLKIGDSHCDKECKAPNQGGSYGYCFKLGCWCEGLPESTPTYPLPDKSCSG-----------------------------------------
 CviNaTBet_Ha1210   KDGYLVKKSDGCKYGCVMLIGDSNCDMECKAPNQGGQKGWCYAFGCWCTGMPESTQVYPLPGKSCGKK----------------------------------------
 CviNaTBet_SCv_01   KEGYLVNKSDGCKYGCVMLIGDEGCNKECKAPNQGGTKGWCYAFGCWCTGMPESTQVYPLPNKSCGKK----------------------------------------
 CviNaTBet_SCv_02   KEGYLVNKSNGCKYGCTPKLGDDHCDTECKARNQGGKKGWCKNFGCWCTGMPESTQTYPIPGKPCSS-----------------------------------------
```
